# Celecoxib‐tramadol co‐crystal in patients with moderate‐to‐severe pain following bunionectomy with osteotomy: A phase 3, randomized, double‐blind, factorial, active‐ and placebo‐controlled trial

**DOI:** 10.1111/papr.13136

**Published:** 2022-07-08

**Authors:** Eugene R. Viscusi, Oscar de Leon‐Casasola, Jesús Cebrecos, Adam Jacobs, Adelaida Morte, Esther Ortiz, Mariano Sust, Anna Vaqué, Ira Gottlieb, Stephen Daniels, Joseph S. Gimbel, Derek Muse, Peter Winkle, Michael E. Kuss, Sebastián Videla, Neus Gascón, Carlos Plata‐Salamán

**Affiliations:** ^1^ Department of Anesthesiology Sidney Kimmel Medical College, Thomas Jefferson University Philadelphia Pennsylvania USA; ^2^ Department of Anesthesiology University of Buffalo/Roswell Park Cancer Institute Buffalo New York USA; ^3^ ESTEVE Pharmaceuticals S.A Barcelona Spain; ^4^ Premier Research Reading UK; ^5^ Chesapeake Research Group LLC Pasadena Maryland USA; ^6^ Optimal Research LLC Austin Texas USA; ^7^ Arizona Research Center Phoenix Arizona USA; ^8^ JBR Clinical Research Salt Lake City Utah USA; ^9^ Anaheim Clinical Trials LLC Anaheim California USA; ^10^ Premier Research Durham North Carolina USA; ^11^ Present address: Michael Kuss Consulting Austin Texas USA; ^12^ Present address: Clinical Research Support Unit Clinical Pharmacology Department Bellvitge University Hospital L’Hospitalet deLlobregat and Pharmacology Unit Department of Pathology and Experimental Therapeutics Faculty of Medicine and Health Sciences IDIBELL, University of Barcelona, L’Hospitalet de Llobregat Barcelona Spain

**Keywords:** acute pain, analgesia, celecoxib, co‐crystal, efficacy, pain, pain, postoperative, postoperative pain, safety, tramadol

## Abstract

**Background:**

Celecoxib‐tramadol co‐crystal (CTC) is a first‐in‐class analgesic co‐crystal of celecoxib and racemic tramadol with an improved pharmacologic profile, conferred by the co‐crystal structure, compared with its active constituents administered alone/concomitantly.

**Aim:**

We evaluated CTC in moderate‐to‐severe acute postoperative pain.

**Materials and Methods:**

This randomized, double‐blind, factorial, active‐ and placebo‐controlled phase 3 trial (NCT03108482) was conducted at 6 US clinical research centers. Adults with moderate‐to‐severe acute pain following bunionectomy with osteotomy were randomized to oral CTC (200 mg [112 mg celecoxib/88 mg rac‐tramadol hydrochloride] every 12 h), tramadol (50 mg every 6 h), celecoxib (100 mg every 12 h), or placebo for 48 h. Patients, investigators, and personnel were blinded to assignment. The primary endpoint was the 0–48 h sum of pain intensity differences (SPID0–48) in all randomized patients. Pain intensity was assessed on a 0–10 numerical rating scale (NRS). Safety was analyzed in patients who received study medication. Funded by ESTEVE Pharmaceuticals.

**Results:**

In 2017 (March to November), 1323 patients were screened and 637 randomized to CTC (n = 184), tramadol (n = 183), celecoxib (n = 181), or placebo (n = 89). Mean baseline NRS was 6.7 in all active groups. CTC had a significantly greater effect on SPID0–48 (least‐squares mean: −139.1 [95% confidence interval: −151.8, −126.5]) than tramadol (−109.1 [−121.7, −96.4]; *p* < 0.001), celecoxib (−103.7 [−116.4, −91.0]; *p* < 0.001), or placebo (−74.6 [−92.5, −56.6]; *p* < 0.001). Total treatment‐emergent adverse events (TEAEs) were 358 for CTC and 394 for tramadol. Drug‐related TEAEs occurred in 37.7% patients in the CTC group, compared with 48.6% in the tramadol group. There were no serious TEAEs/deaths.

**Conclusion:**

CTC provided greater analgesia than comparable daily doses of tramadol and celecoxib, with similar tolerability to tramadol. CTC is approved in the United States.

**What is known**

- There is an unmet need for new multimodal treatment options that are well tolerated, with equal or superior efficacy, and reduced risk of adverse outcomes such as abuse and dependence, compared with current conventional opioid analgesics.
- Today, provision of multimodal analgesia is standard care. For appropriate patients with moderate‐to‐severe acute pain, opioids are often a useful component of multimodal regimens.
- Celecoxib‐tramadol co‐crystal (CTC) has been developed as a multimodal treatment option for the management of acute pain severe enough to require an opioid. CTC contains tramadol, a μ‐opioid agonist and norepinephrine and serotonin reuptake inhibitor, plus the cyclooxygenase‐2 selective inhibitor celecoxib.
- The co‐crystal structure of CTC changes the physicochemical properties of celecoxib and tramadol in a way not achieved by their fixed‐dose or free combination.
- CTC received US Food and Drug Administration approval in October 2021.

**What this study adds**

- Using an established model of acute pain, and comparable doses of active ingredients, the present study demonstrates the superior analgesic efficacy of CTC twice daily (celecoxib 224 mg/*rac*‐tramadol hydrochloride 176 mg) compared with tramadol 50 mg four times daily or celecoxib 100 mg twice daily.
- CTC provided greater efficacy than tramadol or celecoxib, with lower rescue medication use (including significantly less use of rescue opioid), and a safety profile similar to tramadol.

## INTRODUCTION

Celecoxib‐tramadol co‐crystal (CTC) received US Food and Drug Administration approval in October 2021.^1^ CTC is a first‐in‐class analgesic co‐crystal and contains 2 active pharmaceutical ingredients (APIs)—celecoxib and racemic tramadol hydrochloride—in a supramolecular crystal network at a 1:1 molecular ratio. The molecular structure of CTC shows that its three constituent moieties (celecoxib and the two active enantiomers of tramadol) are linked via hydrogen bonding in which chloride ions establish three key intermolecular contacts with adjacent molecules.^2^, ^3^, ^4^ This molecular structure of CTC changes the physicochemical properties of celecoxib and tramadol in a way not achieved by their fixed‐dose or free combination,^4^, ^5^ modifying the pharmacokinetics of each as demonstrated in several clinical crossover studies.^6^, ^7^, ^8^, ^9^ In particular, tramadol from CTC has a lower maximum plasma concentration compared with tramadol taken alone or concomitantly with celecoxib, celecoxib absorption is accelerated with CTC compared with celecoxib alone, and tramadol‐mediated interference of celecoxib absorption is minimized with CTC compared with concomitant administration of celecoxib and tramadol.^6^, ^7^, ^8^ These improved pharmacokinetic profiles have been found to be consistent in females and males, in different races/ethnicities, in fed and fasted conditions, and with single or multiple CTC doses.^6^, ^7^, ^8^, ^9^, ^10^

Combining tramadol (a μ‐opioid agonist and norepinephrine and serotonin reuptake inhibitor) with celecoxib (a cyclooxygenase‐2‐selective inhibitor) in CTC provides multimodal analgesia via 4 complementary mechanisms of action in central and peripheral pain pathways. Multimodal pharmacologic analgesia may improve efficacy beyond that provided by single agents while minimizing dose requirements and dose‐dependent adverse events (AEs) and is recognized as an essential tool in pain management. CTC, therefore, targets an unmet need for efficacious and well‐tolerated multimodal analgesics. CTC also addresses an unmet need for analgesics that are suitable for treating acute pain severe enough to require an opioid and that might also reduce the risk of adverse effects associated with conventional opioids (eg, respiratory depression, abuse, dependence); tramadol has a lower risk of these effects than conventional opioids.^11^, ^12^, ^13^, ^14^, ^15^ Conventional opioids are classified as schedule II drugs by the US Drug Enforcement Administration (DEA), while tramadol is classified as a schedule IV drug. The DEA defines schedule II drugs as those with a high potential for abuse, and schedule IV drugs as those with a low potential for abuse and low risk of dependence, by comparison.^16^

CTC is formulated as a 100‐mg immediate‐release tablet containing 56 mg of celecoxib and 44 mg of tramadol for oral administration. The recommended dosing regimen is 200 mg (112 mg celecoxib/88 mg *rac*‐tramadol hydrochloride) every 12 h as needed for pain relief,^1^ translating to a maximum daily dose of 176 mg of tramadol and 224 mg of celecoxib—approximately half the maximum daily doses of its individual active components. In a phase 2 study, a marked improvement in benefit/risk ratio was observed with CTC versus tramadol and placebo in patients with acute pain after oral surgery.^17^

This phase 3 study evaluated the analgesic efficacy, safety, and tolerability of CTC compared with tramadol, celecoxib, and placebo in patients with moderate‐to‐severe acute pain following bunionectomy with osteotomy. The study followed US Food and Drug Administration (FDA) requirements for the design of factorial studies assessing fixed‐dose combinations.

## METHODS

### Study design

This randomized, double‐blind, factorial, active‐ and placebo‐controlled, parallel‐group, phase 3 clinical trial (NCT03108482) was performed at 6 US clinical research centers. The protocol was approved by an Institutional Review Board; Sterling IRB (Atlanta, GA; IRB ID: 5724). The study was conducted in accordance with the ethical principles originating from the Declaration of Helsinki, the International Council for Harmonization guidelines for Good Clinical Practice, and local regulatory requirements. The protocol and statistical analysis plan are available at https://clinicaltrials.gov/ct2/show/study/NCT03108482. Trial registration (submission) date was March 30, 2017. Patients were screened between March 14 and November 1, 2017; randomization occurred between March 21 and November 7, 2017.

### Patients

Eligible patients were ≥18 years old with moderate‐to‐severe pain (pain intensity rating score of 5–9 on the 0–10 numerical rating scale [NRS]) within 8 h of popliteal sciatic block cessation, following primary unilateral first metatarsal osteotomy with internal fixation and no additional collateral procedure. Full criteria are described in the supplemental digital content. All patients provided written informed consent.

### Randomization and masking

Eligible patients were randomized 2:2:2:1 to oral CTC 200 mg every 12 h, celecoxib 100 mg every 12 h, tramadol 50 mg every 6 h, or placebo every 6 h. Each CTC dose was given as two 100‐mg immediate‐release tablets; each tablet contained 56 mg of celecoxib and 44 mg of racemic tramadol. Randomization was stratified by center and baseline pain score (moderate [NRS 5–6] or severe [NRS 7–9]). At study start, a computer‐generated randomization schedule was prepared and randomization numbers were assigned sequentially via central interactive response technology. No one involved in study conduct had access to the schedule before official unblinding. Patients, investigators, and study personnel were blinded to assignment. To maintain blinding, all medications, including placebo, were provided as over‐encapsulated tablets. In the CTC and celecoxib groups, placebo was administered at the 6‐h midpoint between active treatments.

### Procedures

Study duration was ≤6 weeks, including a screening period of ≤28 days. Patients were admitted to the center on the morning of surgery, remained there for 3 nights (ie, the period for which rescue medication could be administered), and returned for a follow‐up visit 5 to 9 days post‐surgery. The treatment period was 48 h.

Patients underwent bunionectomy with osteotomy and internal fixation under standardized regional anesthetic, involving popliteal sciatic nerve block and continuous sciatic infusion via a catheter placed in the proximity of the popliteal sciatic nerve. Patients received midazolam and/or propofol for initial sedation, lidocaine 1% for local anesthesia, and ropivacaine 0.5% for popliteal sciatic nerve block. If popliteal sciatic nerve block was insufficient to provide adequate anesthesia, a standard Mayo block with lidocaine 2% was permitted. Regional anesthesia (mepivacaine 0.5% or ropivacaine 0.2%) was continued after surgery, via continuous infusion, and discontinued between 3:00 and 4:00 am the day following surgery. After this time, patients meeting the inclusion criteria for baseline pain intensity were randomly assigned to treatment. Supplemental analgesia with intravenous ketorolac was permitted until 1:00 am to help control breakthrough pain if the regional anesthetic infusion was insufficient to provide adequate analgesia.

Patients could receive rescue medication for pain after discontinuation of anesthetic and initiation of study medication (first line: acetaminophen [paracetamol], 1 g intravenously every 4–6 h as needed, up to 4 g in 24 h; second line [if acetaminophen not tolerated/provided insufficient analgesia]: oxycodone, 5‐mg immediate‐release tablets orally every 4–6 h as needed, up to 30 mg in 24 h). Patients were encouraged to wait ≥1 h after the first dose of study medication before receiving rescue medication.

Pain intensity was assessed using the NRS (0–10; 0 = no pain, 10 = worst possible pain) at: 0 h (before study medication); 15, 30, and 45 min; 1, 1.25, 1.5, 1.75, 2, 2.5, 3, 3.5, 4, 5, 6, 7, 8, 10, 12, 14, 18, 22, 24, 26, 28, 30, 32, 34, 36, 38, 42, 46, and 48 h; and immediately before rescue medication. Pain relief scores were assessed at the same time points, using a 5‐point categorical scale. Patients were asked, “How much relief have you had since your starting pain?”, with response choices of none (score of 0), a little (1), some (2), a lot (3), and complete (4). The pain intensity assessment was completed before the pain relief assessment; patients were not permitted to compare their responses with previous responses. Pain intensity and pain relief results were recorded in patient diaries. Pain assessments were performed during the night only if the patient was awake (patients were awakened to administer study medication). Time to perceptible and meaningful pain relief was measured using the double stopwatch technique starting at time 0 (ie, immediately after the patient swallowed the last tablet of the first dose of study medication). Patients were instructed to stop the first stopwatch when they perceived any pain relief, and to stop the second stopwatch when they experienced pain relief that was meaningful to them. Patients provided an overall assessment of study medication by answering the following global evaluation question at the end of the 48‐h treatment period: “How effective do you think the study medication is as a treatment for pain?” (response choices were 0 = poor, 1 = fair, 2 = good, 3 = very good, and 4 = excellent). Safety and tolerability were evaluated until follow‐up based on the incidence, severity, and relationship of treatment‐emergent adverse events (TEAEs) to study medication, physical examination findings, clinical laboratory test results, and changes in vital sign and electrocardiogram measurements. TEAEs were defined as AEs with onset at the time of or following the start of study medication, or starting beforehand but increasing in severity or relationship at the time of or following the start of study medication, occurring up to 7 days after the final dose of study medication.

### Efficacy outcomes

The primary efficacy variable was pain intensity measured by NRS. The primary efficacy endpoint was the sum of pain intensity differences (SPID) from 0 to 48 h (SPID_0–48_). SPID was calculated as a time‐weighted sum of pain intensity difference values at each follow‐up time point (difference between starting pain intensity and pain intensity at the given assessment time point) multiplied by time (h) since the last non‐missing assessment.

Secondary efficacy endpoints included SPID over 4, 6, 12, and 24 h; total pain relief (TOTPAR; time‐weighted sum of pain relief scores at each time point) over 4, 6, 12, 24, and 48 h; pain intensity and change from baseline in pain intensity at each time point; response rates; time to onset of analgesia (defined as time to perceptible pain relief by stopwatch assessment, if confirmed by meaningful pain relief by stopwatch assessment within 8 h); proportion of patients using rescue medication; time to first use of rescue medication; and patients' overall assessment of study drug. Post hoc analyses on use of oxycodone rescue medication and on placebo‐adjusted efficacy (SPID_0–48_) were performed, the latter to permit estimation of the absolute efficacy of CTC.

### Statistical analyses

The study aimed to demonstrate superiority of CTC 200 mg over tramadol and celecoxib. To determine sample size, power for both comparisons of CTC (vs tramadol and vs celecoxib) was set at 90%; therefore, overall study power was 81%. To detect a difference in SPID_0–48_ of 48 units, assuming a standard deviation (SD) of 124, at a 2‐sided alpha level of 0.05 and 90% power, 142 patients per group were required (71 for placebo). Allowing for a 20% rate of nonevaluable patients, 180 individuals per group (90 for placebo) were to be randomized—630 in total. For sample size re‐estimation, a planned blinded interim analysis was performed after about half the patients had completed the study. In this analysis, estimated pooled variance of the primary outcome measure was substantially lower than that used for sample size calculation; thus, it was concluded that the study was adequately powered and there was no need to increase sample size.

The full analysis set comprised all randomized patients. The per‐protocol analysis included all patients in the full analysis set with no major protocol deviations. The safety analysis included all patients who received study medication. Efficacy analyses were performed on the full analysis set, sensitivity analyses on the per‐protocol analysis set, and safety analyses on the safety analysis set.

The primary efficacy analysis compared SPID_0–48_ across treatment groups using an analysis of covariance (ANCOVA) model, adjusting for center and baseline pain intensity. To account for use of rescue medication, a pain intensity assessment was performed upon each request for rescue medication, with the score used to replace all scores for timed assessments taken in the subsequent 4 h (considered to be the period over which rescue medication was active). ANCOVA analyses were performed for all quantitative efficacy outcomes; *p*‐values and 95% confidence intervals (CIs) for the least‐squares (LS) means for pairwise treatment differences were derived.

An additional analysis of pain intensity difference from baseline by time point was conducted using a mixed model for repeated measures analysis on the full analysis set using type 3 tests of fixed effects.

The proportion of patients requiring rescue medication and of responders, and patients' overall assessment of study drug, were analyzed by logistic regression, adjusted by baseline pain and center. Time to onset of analgesia and first use of rescue medication were analyzed using time‐to‐event statistical methods. Safety data were analyzed and reported using descriptive statistics.

Additional sensitivity analyses were conducted to assess the impact of rescue medication use and missing data imputation due to study discontinuation. Efficacy analyses were also performed by subgroups defined by baseline pain intensity, sex, age, body mass index (BMI), and race.

All statistical analyses were conducted using SAS 9.4 (SAS Institute Inc., Cary, NC).

## RESULTS

**FIGURE 1:**
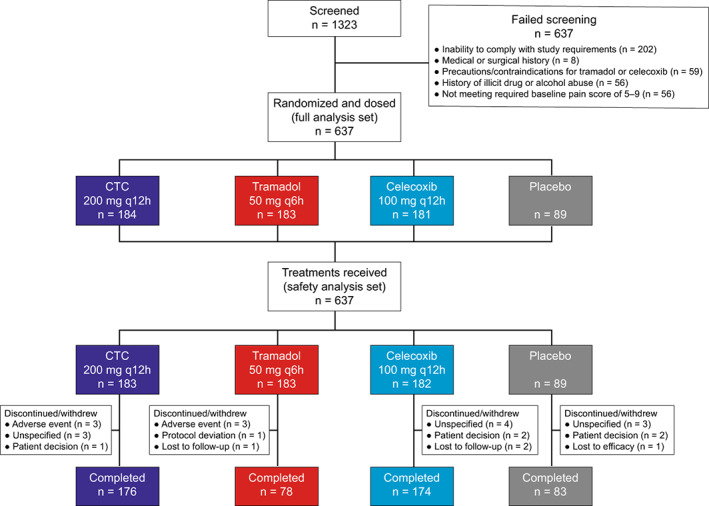
Patient disposition. CTC, celecoxib‐tramadol co‐crystal; q6h, every 6 h; q12h, every 12 h

Of 1323 patients screened, 637 were randomized to CTC (*n* = 184), tramadol (*n* = 183), celecoxib (*n* = 181), or placebo (*n* = 89) (Figure 1). One patient with severe pain was randomized to CTC but received celecoxib in error. This patient was included in the CTC group in the full analysis set following an intent‐to‐treat principle; safety data were analyzed according to treatment received (celecoxib). Twenty‐six patients in the safety analysis set (CTC, *n* = 7; tramadol, *n* = 5; celecoxib, *n* = 8; placebo, *n* = 6) discontinued treatment or withdrew from the study.

Baseline characteristics were similar across groups, with most patients being female (86%) and white (74%). Mean age was 46 years, mean body weight was 76 kg, and mean BMI was 28 kg/m^2^ (Table 1). Mean (SD) baseline NRS pain score was 6.7 (1.33). Similar proportions of patients reported moderate or severe pain across groups.

**TABLE 1:** Baseline characteristics (full analysis set)

	CTC	Tramadol	Celecoxib	Placebo
Sample size, *n*	184	183	181	89
Age (y)	43.9 (14.4)	48.1 (14.4)	45.1 (13.0)	46.1 (14.9)
<65 y, *n* (%)	173 (94.0)	159 (86.9)	165 (91.2)	82 (92.1)
≥65 y, *n* (%)	11 (6.0)	24 (13.1)	16 (8.8)	7 (7.9)
Sex, *n* (%)				
Female	162 (88.0)	150 (82.0)	160 (88.4)	75 (84.3)
Male	22 (12.0)	33 (18.0)	21 (11.6)	14 (15.7)
Race, *n* (%)				
White	138 (75.0)	128 (69.9)	138 (76.2)	66 (74.2)
Black or African American	42 (22.8)	40 (21.9)	33 (18.2)	20 (22.5)
Asian	2 (1.1)	14 (7.7)	8 (4.4)	3 (3.4)
Native Hawaiian/Pacific Islander	5 (2.7)	1 (0.5)	1 (0.6)	1 (1.1)
American Indian/Alaska Native	1 (0.5)	2 (1.1)	2 (1.1)	0
Other	1 (0.5)	1 (0.5)	2 (1.1)	0
Ethnicity, *n* (%)				
Not Hispanic/Latino	134 (72.8)	138 (75.4)	143 (79.0)	71 (79.8)
Hispanic/Latino	50 (27.2)	45 (24.6)	38 (21.0)	18 (20.2)
Weight (kg)	76.7 (16.1)	75.0 (15.7)	76.4 (16.9)	76.3 (15.8)
BMI (kg/m^2^)	28.1 (5.0)	27.6 (4.9)	27.9 (5.1)	27.8 (5.2)
Bunionectomy procedure time intervals				
Surgery duration (min)	31.0 (12.6)	30.0 (11.6)	31.2 (12.0)	31.5 (13.2)
Interval from end of surgery to PSB cessation (h)	16.8 (2.4)	16.8 (2.7)	16.8 (2.7)	16.8 (2.6)
Interval from PSB cessation to first treatment (h)	2.6 (2.2)	2.6 (2.2)	2.5 (2.3)	2.8 (2.3)
Baseline pain intensity, NRS	6.7 (1.3)	6.7 (1.4)	6.7 (1.4)	6.8 (1.3)
Baseline pain scores, *n* (%)				
Moderate (NRS 5–6)	89 (48.4)	90 (49.2)	86 (47.5)	42 (47.2)
Severe (NRS 7–9)	95 (51.6)	93 (50.8)	95 (52.5)	47 (52.8)

*Note*: Data reported as mean (SD) unless indicated otherwise.

Abbreviations: BMI, body mass index; CTC, celecoxib‐tramadol co‐crystal; NRS, numerical rating scale; PSB, popliteal sciatic block; SD, standard deviation.

For SPID_0–48_, CTC twice daily (celecoxib 224 mg/tramadol 176 mg) provided a significantly greater analgesic effect than tramadol 50 mg administered 4 times daily (LS mean difference: –30.0, *p <* 0.001), celecoxib 100 mg twice daily (−35.4, *p <* 0.001), and placebo (−64.6, *p <* 0.001; Table 2). Tramadol and celecoxib provided significantly greater analgesia (measured by SPID_0–48_) versus placebo (LS mean difference: −34.53 and −29.14, respectively, *p <* 0.01).

**TABLE 2:** SPID and TOTPAR (full analysis set)

	LS mean (95% CI)				LS mean difference (95% CI) [*p*‐value]		
	CTC (*n* = 184)	Tramadol (*n* = 183)	Celecoxib (*n* = 181)	Placebo (*n* = 89)	CTC versus tramadol	CTC versus celecoxib	CTC versus placebo
Primary endpoints^a^							
SPID 0–48 h	−139.1 (−151.8, −126.5)	−109.1 (−121.7, −96.4)	−103.7 (−116.4, −91.0)	−74.6 (−92.5, −56.6)	−30.0 (−47.5, −12.6) [*p* < 0.001]	−35.4 (−52.9, −18.0) [*p* < 0.001]	−64.6 (−86.1, −43.0) [*p* < 0.001]
Secondary endpoints^a^							
SPID 0–4 h	−5.1 (−6.4, −3.9)	−3.7 (−5.0, −2.4)	−2.7 (−4.0, −1.5)	−0.2 (−2.0, 1.6)	−1.4 (−3.2, 0.3) [*p* = 0.11]	−2.4 (−4.2, −0.6) [*p* < 0.01]	−5.0 (−7.1, −2.8) [*p* < 0.001]
SPID 0–6 h	−8.1 (−9.9, −6.4)	−5.1 (−6.8, −3.3)	−4.5 (−6.3, −2.8)	−0.3 (−2.8, 2.1)	−3.1 (−5.5, −0.7) [*p* = 0.01]	3.6 (−6.0, −1.2) [*p* < 0.01]	−7.8 (−10.8, −4.8) [*p* < 0.001]
SPID 0–12 h	−18.0 (−21.0, −14.9)	−12.7 (−15.8, −9.7)	−11.2 (−14.3, −8.2)	−3.3 (−7.6, 1.1)	−5.2 (−9.4, −1.0) [*p* = 0.01]	−6.7 (−10.9, −2.5) [*p* < 0.01]	14.7 (−19.9, −9.5) [*p* < 0.001]
SPID 0–24 h	−51.4 (−57.5, −45.3)	−37.8 (−43.8, −31.7)	−33.4 (−39.5, −27.3)	−15.4 (−24.0, −6.8)	−13.6 (−22.0, −5.3) [*p* < 0.01]	−18.0 (−26.4, −9.6) [*p* < 0.001]	−36.0 (−46.4, −25.7) [*p* < 0.001]
TOTPAR 0–4 h^b^	4.8 (4.1, 5.4)	4.0 (3.3, 4.6)	3.3 (2.7, 3.9)	2.5 (1.6, 3.3)	0.8 (−0.05, 1.6) [*p* = 0.07]	1.5 (0.6, 2.3) [*p* < 0.001]	2.3 (1.2, 3.3) [*p* < 0.001]
TOTPAR 0–6 h^b^	7.4 (6.6, 8.3)	6.0 (5.2, 6.9)	5.1 (4.3, 5.9)	3.7 (2.5, 4.9)	1.4 (0.2, 2.5) [*p* = 0.02]	2.3 (1.2, 3.5) [*p* < 0.001]	3.7 (2.3, 5.1) [*p* < 0.001]
TOTPAR 0–12 h^b^	16.1 (14.6, 17.5)	13.9 (12.4, 15.4)	11.7 (10.2, 13.2)	9.2 (7.1, 11.3)	2.2 (0.1, 4.2) [*p* = 0.04]	4.4 (2.3, 6.4) [*p* < 0.001]	6.9 (4.4, 9.4) [*p* < 0.001]
TOTPAR 0–24 h^b^	39.4 (36.4, 42.5)	34.3 (31.2, 37.4)	29.2 (26.1, 32.3)	23.5 (19.1, 27.8)	5.1 (0.9, 9.3) [*p* = 0.02]	10.2 (6.0, 14.5) [*p* < 0.001]	15.9 (10.7, 21.2) [*p* < 0.001]
TOTPAR 0–48 h^b^	94.3 (87.7, 100.8)	83.0 (76.5, 89.6)	76.1 (69.5, 82.6)	64.3 (55.0, 73.5)	11.2 (2.2, 20.2) [*p* = 0.01]	18.2 (9.2, 27.2) [*p* < 0.001]	30.0 (18.9, 41.2) [*p* < 0.001]

Abbreviations: CI, confidence interval; CTC, celecoxib‐tramadol co‐crystal; LS, least‐squares; SPID, sum of pain intensity difference; TOTPAR, total pain relief.

^a^ Analyzed using analysis of covariance, adjusting for center and baseline pain intensity.

^b^ Pain relief was measured from 0.25 h, using time 0 as reference.

Sensitivity analyses assessing the impact of adjustments for rescue medication and missing data imputation supported primary efficacy findings (Table S1). The estimated placebo‐adjusted treatment effect of CTC on SPID_0–48_ was approximately 1.9 and 2.2 times larger than that of tramadol and celecoxib, respectively (Figure S1).

Findings of a subgroup analysis of SPID_0–48_ by baseline pain were generally consistent across subgroups and with the primary efficacy analysis (Figure S2). In patients with severe pain, CTC provided a significantly greater effect versus tramadol (LS mean difference: –36.75, *p <* 0.01), celecoxib (−25.86, *p* = 0.04), and placebo (−71.23, *p <* 0.001). In patients with moderate pain, CTC provided a significantly greater effect versus celecoxib (−46.05, *p <* 0.001) and placebo (−57.31, *p <* 0.001); the comparison versus tramadol was not statistically significant (−23.02, *p* = 0.07). Results of subgroup analyses by sex, age, and BMI generally mirrored those of the main analyses, although treatment differences did not always reach statistical significance, particularly in smaller subgroups for which statistical power was low (Figure S1).

**FIGURE 2:**
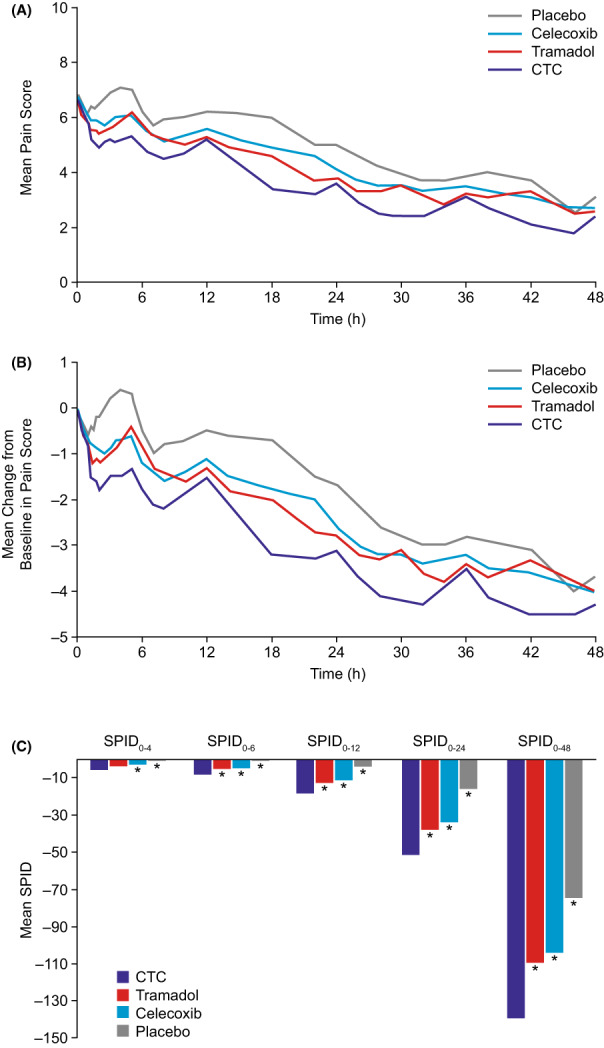
(A) Mean pain intensity (NRS score) over time. (B) Mean change from baseline in pain intensity (NRS score) over time. (C) Sum of pain intensity differences (SPID) by time point. Full analysis set; data are adjusted for rescue medication. Analyzed using analysis of covariance, adjusting for center and baseline pain intensity. Asterisk (*) denotes *p* < 0.05 versus CTC. CTC, celecoxib‐tramadol co‐crystal; NRS, numerical rating scale

Mean pain intensity scores in active treatment groups decreased and separated shortly after first administration of study drug (Figure 2A). Mean changes from baseline in pain intensity were greater in the CTC group than in other groups at all time points (Figure 2B). Reductions in pain intensity difference from baseline became statistically significant for CTC over placebo at 1 h (LS mean: –0.74, *p* = 0.01), over celecoxib at 1.25 h (−0.65, *p* = 0.01), and over tramadol at 3.5 h (−0.55, *p* = 0.04). Analyses of SPID and TOTPAR at 4, 6, 12, and 24 h and TOTPAR at 48 h were consistent with primary endpoint findings (Table S1; Figure 2C). For SPID and TOTPAR, CTC reached statistical significance versus tramadol from 6 h and versus celecoxib and placebo from 4 h (Table 2).

The highest proportion of responders was in the CTC group, although an overall majority of study patients were classified as responders, and variations in response rates between groups were not significantly different (Table S2).

**FIGURE 3:**
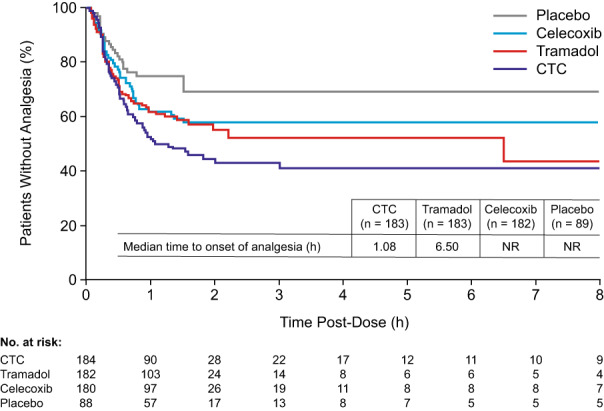
Onset of analgesia (time to onset and proportion of patients). Full analysis set. Time to onset of analgesia was defined as the time to perceptible pain relief by stopwatch assessment, if confirmed by meaningful pain relief by stopwatch assessment within 8 h. CTC, celecoxib‐tramadol co‐crystal; NR, not reached

Median time to onset of analgesia was 1.08 h for CTC and 6.5 h for tramadol (hazard ratio [HR] = 1.293, 95% CI: 0.959, 1.743; Figure 3). Median time to onset of analgesia was not reached for celecoxib or placebo, as less than half of patients achieved analgesia (HRs [95% CI] versus CTC = 1.408 [1.037, 1.913] and 2.238 [1.432, 3.499], respectively).

**FIGURE 4:**
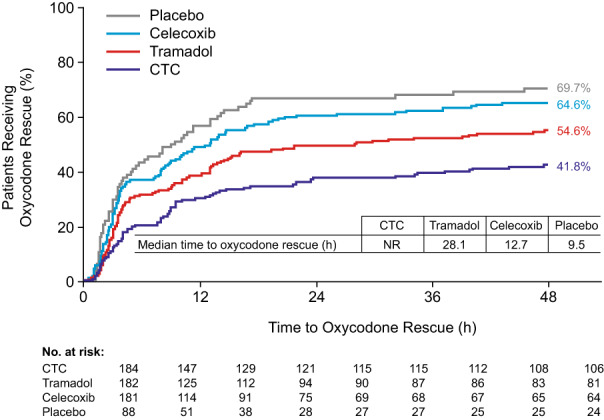
Use of oxycodone rescue medication (time to first use and proportion of patients). Odds ratio for oxycodone rescue medication use at 48 h for CTC versus tramadol: 0.581 (95% confidence interval: 0.378, 0.891; *p* = 0.01). Logistic regression adjusted for center and baseline pain. CTC, celecoxib‐tramadol co‐crystal; NR, not reached

Most patients used first‐line rescue pain medication. Around half used second‐line, opioid rescue pain medication. Fewer patients in the CTC group than in other groups required rescue medication (Table 3; similar results were obtained for the use of intravenous acetaminophen, given this was first line); patients in the CTC group used rescue medication later and took fewer doses (Table 3). Most patients in the CTC group (58.2%) did not require oxycodone, with lower use in this group evident from 6 h post‐dose and sustained to 48 h (vs tramadol, celecoxib, and placebo); median time to oxycodone use was not reached for CTC (Table 3; Figure 4).

**TABLE 3:** Rescue medication use and patient global evaluation of study drug (full analysis set)

	Treatment group				Hazard ratio or odds ratio (95% CI) [*p*‐value]		
	CTC (*n* = 184)	Tramadol (*n* = 183)	Celecoxib (*n* = 181)	Placebo (*n* = 89)	CTC versus tramadol	CTC versus celecoxib	CTC versus placebo
Median time to first rescue medication (h)							
Any rescue medication	4.2	2.2	2.1	1.6	HR = 0.714^b^ (0.567, 0.899) [*p* < 0.01]	HR = 0.657^b^ (0.523, 0.826) [*p* < 0.001]	HR = 0.536^b^ (0.406, 0.709) [*p* < 0.001]
Oxycodone^a^	NR	28.1	12.7	9.5	HR = 0.675^b^ (0.501, 0.910) [*p* < 0.01]	HR = 0.510^b^ (0.382, 0.681) [*p* < 0.001]	HR = 0.432^b^ (0.308, 0.605) [*p* < 0.001]
Patients receiving rescue medication, *n* (%)							
Within 12 h							
Any rescue medication	132 (71.7)	148 (80.9)	152 (84.0)	76 (85.4)	–	–	–
Oxycodone	55 (29.9)	71 (38.8)	90 (49.7)	50 (56.2)	–	–	–
Within 48 h							
Any rescue medication	140 (76.1)	153 (83.6)	160 (88.4)	79 (88.8)	OR = 0.598 (0.351, 1.018)^c^ [*p* = 0.06]	OR = 0.398 (0.223, 0.713)^c^ [*p* < 0.01]	OR = 0.396 (0.186, 0.843)^c^ [*p* = 0.02]
Oxycodone	77 (41.8)	100 (54.6)	117 (64.6)	62 (69.7)	OR = 0.581 (0.378, 0.891)^c^ [*p* = 0.01]	OR = 0.372 (0.240, 0.577)^c^ [*p* < 0.001]	OR = 0.297 (0.170, 0.519)^c^ [*p* < 0.001]
Doses of rescue medications, *n* (%)							
Any rescue medication dose							
0	44 (23.9)	30 (16.4)	21 (11.6)	10 (11.2)	OR = 0.532 (0.370, 0.766)^d^ [*p* < 0.001]	OR = 0.387 (0.267, 0.559)^d^ [*p* < 0.001]	OR = 0.278 (0.176, 0.439)^d^ [*p* < 0.001]
1–2	76 (41.3)	51 (27.9)	50 (27.6)	22 (24.7)			
≥3	64 (34.8)	102 (55.7)	110 (60.8)	57 (64.0)			
Oxycodone rescue medication dose^a^							
0	107 (58.2)	83 (45.4)	64 (35.4)	27 (30.3)	OR = 0.633 (0.427, 0.939)^d^ [*p* = 0.02]	OR = 0.382 (0.258, 0.565)^d^ [*p* < 0.001]	OR = 0.262 (0.163, 0.420)^d^ [*p* < 0.001]
1–2	47 (25.6)	64 (35.0)	62 (34.3)	23 (25.8)			
≥3	30 (16.3)	36 (19.7)	55 (30.4)	39 (43.8)			
Patient global evaluation, *n* (%)^d^							
Good, very good, or excellent	144 (78.3)	128 (70.0)	108 (59.7)	43 (48.3)	OR = 1.358 (0.938, 1.966)	OR = 2.371 (1.633, 3.442)	OR = 3.954 (2.475, 6.317)
Fair	17 (9.2)	27 (14.8)	30 (16.6)	14 (15.7)			
Poor	22 (12.0)	25 (13.7)	43 (23.8)	30 (33.7)			

Abbreviations: CI, confidence interval; CTC, celecoxib‐tramadol co‐crystal; HR, hazard ratio; NR, not reached; OR, odds ratio.

^a^ Post hoc analysis.

^b^ Cox proportional hazards regression adjusted for center and baseline pain.

^c^ Logistic regression adjusted for center and baseline pain.

^d^ Ordinal logistic regression adjusted for center and baseline pain.

Of patients treated with CTC, 78.3% (⁓4/5) reported that analgesia was good, very good, or excellent, versus 70.0% for tramadol, 59.7% for celecoxib, and 48.3% for placebo (Table 3).

Table 4 summarizes the safety data. The proportion of patients reporting any TEAE was 63.4% in both CTC and tramadol groups, 52.2% in the celecoxib group, and 57.3% in the placebo group. Few TEAEs were considered severe. There were no serious TEAEs or deaths. Six patients experienced TEAEs leading to study medication discontinuation: 3 in the CTC group (nausea, *n* = 2; pruritus and rash, *n* = 1) and 3 in the tramadol group (vomiting, *n* = 2; supraventricular tachycardia, *n* = 1); all events resolved. The most common TEAEs were nausea, dizziness, vomiting, and headache. No safety concerns were observed in laboratory tests, electrocardiograms, or vital sign assessments.

**TABLE 4:** Summary of TEAEs from enrollment to follow‐up at 5 to 9 days post‐surgery (safety analysis set)

	CTC	Tramadol	Celecoxib	Placebo
Sample size	183	183	182	89
Total TEAEs	358	394	222	122
Patients with ≥1 TEAE	116 (63.4)	116 (63.4)	95 (52.2)	51 (57.3)
Patients with drug‐related TEAE	69 (37.7)	89 (48.6)	40 (22.0)	22 (24.7)
Patients with TEAE by severity				
Mild	73 (39.9)	64 (35.0)	70 (38.5)	34 (38.2)
Moderate	41 (22.4)	48 (26.2)	23 (12.6)	15 (16.9)
Severe	2 (1.1)	4 (2.2)	2 (1.1)	2 (2.2)
Patients with serious TEAE	0	0	0	0
Patients with TEAE leading to discontinuation	3 (1.6)	3 (1.6)	0	0
Most common TEAE occurring in ≥5% of patients in any group				
Nausea	55 (30.1)	69 (37.7)	30 (16.5)	17 (19.1)
Dizziness	31 (16.9)	34 (18.6)	9 (4.9)	13 (14.6)
Vomiting	29 (15.8)	30 (16.4)	4 (2.2)	2 (2.2)
Headache	21 (11.5)	33 (18.0)	20 (11.0)	6 (6.7)
Somnolence	15 (8.2)	10 (5.5)	4 (2.2)	3 (3.4)
Decreased appetite	6 (3.3)	11 (6.0)	1 (0.5)	0
Constipation	4 (2.2)	13 (7.1)	9 (4.9)	3 (3.4)

*Note*: Data reported as *n* or *n* (%).

Abbreviations: CTC, celecoxib‐tramadol co‐crystal; TEAE, treatment‐emergent adverse event.

## DISCUSSION

We evaluated the efficacy of CTC, a novel API–API co‐crystal containing celecoxib and tramadol, for the management of moderate‐to‐severe postoperative pain following bunionectomy with osteotomy. CTC was approved by the FDA in October 2021 and, to our knowledge, is the first API–API co‐crystal for analgesia to be specifically acknowledged as such within the prescribing information.^1^ The aim of employing co‐crystal technology is to improve upon the properties of constituent APIs as single entities. In the present study, CTC demonstrated statistically significantly greater analgesic effects on SPID_0–48_ than comparable daily doses of tramadol or celecoxib, and placebo, thereby meeting the study's primary endpoint. SPID_0–48_ was selected as the study's primary efficacy endpoint as it provides a summary of treatment effects on pain intensity over a clinically meaningful time period. Results of sensitivity analyses using different imputation procedures for missing data and rescue medication use, and of a placebo‐adjusted analysis of treatment effects on SPID_0–48_, were consistent with those of the primary analysis. Subgroup analyses for the primary endpoint (conducted by baseline pain, sex, age, race, and BMI) were also generally consistent, although treatment differences did not always reach statistical significance, particularly in smaller subgroups.

Secondary efficacy endpoints supported primary findings, with most reaching statistical significance in favor of CTC. Reductions in mean pain intensity difference from baseline were statistically significantly greater for CTC over placebo, celecoxib, and tramadol at 1, 1.25, and 3.5 h post‐dose, respectively. Due to study medication, rescue medication, and/or natural post‐surgery resolution of pain, most study patients were classified as responders at some point during the 48‐h dosing period; response rates were not significantly different between groups but were highest in the CTC group. Most patients used first‐line rescue pain medication (intravenous acetaminophen), and about half used second‐line (oxycodone). Patients in the CTC group used less rescue medication, and used it later, than patients in other groups. Most patients in the CTC group (58%) did not require oxycodone, compared with 30% to 45% (under half) in other groups, suggesting that CTC may have an opioid‐sparing effect while improving overall analgesia versus other treatments.

The overall rate of TEAEs was similar in the CTC and tramadol groups and lower in the celecoxib and placebo groups. The most common TEAEs were consistent with known safety profiles of tramadol and were as expected in the acute postoperative setting. TEAEs included all events occurring from enrollment to follow‐up and, therefore, captured AEs arising from confounding factors (eg, concomitant/rescue medications, medical procedures). In phase 1 studies designed to avoid such factors, AE rates were lower with CTC than with tramadol alone, or with tramadol and celecoxib in free combination.^6^, ^8^ For example, in the phase 1 study that served as a bridging study for this phase 3 trial, drug‐related TEAEs were experienced by a lower proportion of participants receiving CTC 200 mg (6%) than for tramadol 100 mg (19%) or for the free combination of celecoxib 100 mg and tramadol 100 mg (21%).^6^

This study used an established model of acute surgical pain (bunionectomy with osteotomy) associated with pain of sufficient intensity to warrant opioid use, and of sufficient duration to measure efficacy over multiple days; the model provides good sensitivity for approximately 72 h post‐surgery.^18^, ^19^, ^20^ Thus, findings are generalizable to the wider population of adult patients with pain severe enough to require an opioid where alternatives are inadequate, a proposition supported by the recent US regulatory approval of CTC.^1^ Findings are consistent with a phase 2 study, in which patients with moderate‐to‐severe pain following oral surgery with bone removal received single doses of CTC, tramadol, or placebo.^17^ Clinical evidence to date suggests that CTC's unique modification of its therapeutic moieties, plus their complementary and synergistic central and peripheral mechanisms of action, may impart enhanced therapeutic benefit for acute pain, ultimately permitting efficacy at lower doses than recommended for its constituents.

Given ongoing problems with opioid misuse and abuse,^21^ there are medical and public health needs for efficacious analgesics with lower abuse potential than conventional opioids.^11^ In the United States, access to treatments for relief of moderate‐to‐severe pain can be challenging, given growing restrictions on prescription and use of conventional opioids (classified as schedule II controlled substances by the US DEA). CTC contains tramadol, a schedule IV opioid in the United States, which is associated with a lower potential for abuse and dependence than schedule II opioids.^11^, ^12^, ^16^, ^22^ CTC doses used in this study (200 mg every 12 h) exposed patients to 176 mg/day of tramadol, corresponding to 17.6 morphine milligram equivalents (MME), below the recommended threshold of 20 to 50 MME/day for a relatively low‐dose opioid.^23^, ^24^ Additionally, although there is a risk of serotonin syndrome in certain patient subpopulations, tramadol is generally associated with a lower risk of opioid‐related side effects, such as respiratory depression and constipation, than conventional opioids.^15^, ^25^ Due to these factors, and the benefit/risk profile reported here and previously,^17^ CTC may provide an alternative option for appropriately selected adult patients who would otherwise receive a conventional (schedule II) opioid.

The recommended CTC dose translates into a maximum daily dose of 224 mg of celecoxib and 176 mg oftramadol—about half the maximum daily doses of its individual active components based on the full prescribing information of celecoxib and tramadol for acute pain.^26^, ^27^ Chronic use of high doses of celecoxib (≥400 mg) has been associated with an increased risk of cardiovascular events^28^; however, at moderate doses (≤200 mg), celecoxib is noninferior to ibuprofen and naproxen for cardiovascular safety.^29^ Acute exposure to celecoxib associated with short‐term administration of CTC (ie, 224 mg/day of celecoxib) is unlikely to be associated with the increased risk of cardiovascular events reported with chronic use of higher celecoxib doses. Moreover, CTC 200 mg is not associated with higher celecoxib exposure than celecoxib 100 mg alone.^1^, ^6^

The study represents a well‐designed and fair comparison between CTC and its individual APIs. However, possible limitations should be acknowledged. First, doses of tramadol and celecoxib used in comparator groups may have been lower than full therapeutic doses. Although differences in tramadol and celecoxib dosage between CTC and comparator groups mean that the study might not be a true factorial study, the FDA considered that the study met requirements for a well‐controlled, full‐factorial, phase 3 study and proposed the dosing used (the 1:1 molecular ratio of tramadol to celecoxib in CTC is defined by the intrinsic chemical structure, which is equivalent to a 1:1.27 weight ratio of tramadol to celecoxib; thus, CTC 100 mg comprises 44 mg tramadol and 56 mg celecoxib). Second, most patients enrolled were female, although this is typical for the clinical population seeking treatment for bunionectomy. Subgroup analysis of the primary endpoint by sex showed no significant interactions, albeit this analysis was not well powered due to the small number of males. Third, the study did not include a head‐to‐head comparison versus the individual reference products taken together. However, earlier data show that the pharmacokinetics of the APIs in CTC are different to those observed when commercially available celecoxib and tramadol are administered in free combination.^6^, ^8^ In phase 1 studies, the free combination markedly reduced celecoxib absorption, which was not observed with the use of CTC. Administration of CTC was instead associated with slower absorption and lower maximum plasma concentration of tramadol.^6^, ^8^ These differences are likely due to changes in physicochemical properties imparted by the crystalline structure and unique heteromolecular interactions within CTC (ie, hydrogen and ionic bonding)—intrinsic dissolution rate studies have shown that tramadol release from CTC is reduced, while that of celecoxib is increased, versus tramadol or celecoxib alone or their combination^4^, ^5^—and their translation to human pharmacokinetics.^6^, ^8^ For example, in the crossover bridging phase 1 study, which used the same reference products as this phase 3 trial, mean maximum plasma concentration (ng/ml) of tramadol was 214, 305, and 312 with CTC, tramadol alone, and a free combination of tramadol and celecoxib, respectively. For celecoxib, the mean was 259, 318, and 165 with CTC, celecoxib alone, and the free combination, respectively.^1^, ^6^ These differences in pharmacokinetics likely underlie observations in the current study: early onset of analgesia with CTC may be attributable to its celecoxib component (which, in the phase 1 bridging study, had a median *T*max [h] of 1.5, 3.0, and 2.5 from CTC, celecoxib alone, and free combination with tramadol, respectively^1^, ^6^) while modifications in tramadol pharmacokinetics may contribute to its favorable tolerability profile. Total daily doses of celecoxib and tramadol in CTC are considerably lower than the individual doses of celecoxib and tramadol typically prescribed for acute pain (224 mg and 176 mg in CTC vs ≥400 mg and 400 mg for celecoxib and tramadol, respectively). Taken together, these findings suggest that the clinical effects of CTC would not be replicated by concomitant administration of its components or by a fixed‐dose combination; that is, that CTC could not be substituted by either of these approaches.

In conclusion, the API–API co‐crystal CTC provided greater analgesic efficacy than did comparable daily doses of tramadol and celecoxib alone. This was accompanied by lower utilization of rescue pain medication, including significantly lower use of opioid rescue medication. The safety profile of CTC 200 mg (112 mg celecoxib/88 mg tramadol) administered every 12 h was similar to that of tramadol 50 mg administered every 6 h. Our findings suggest that CTC could be an efficacious and well‐tolerated alternative for appropriately selected adult patients with acute pain severe enough to require an opioid, by providing multimodal analgesia with an anti‐inflammatory component, via a means not replicated by free or fixed‐dose combination.

## AUTHOR CONTRIBUTIONS

E.R. Viscusi, O. de Leon‐Casasola, J. Cebrecos, A. Jacobs, A. Morte, M. Sust, A. Vaqué, M.E. Kuss, S. Videla, N. Gascón, and C. Plata‐Salamán conceived or designed the study. J. Cebrecos, E. Ortiz, I. Gottlieb, S. Daniels, J.S. Gimbel, D. Muse, P. Winkle, and S. Videla contributed to data collection. J. Cebrecos, A. Jacobs, E. Ortiz, and M. Sust analyzed the data. E.R. Viscusi, O. de Leon‐Casasola, J. Cebrecos, A. Jacobs, A. Morte, M. Sust, A. Vaqué, I. Gottlieb, M.E. Kuss, and C. Plata‐Salamán interpreted the data. All authors drafted, edited, or reviewed drafts of the manuscript and approved the final version. All authors had access to the underlying data. E.R. Viscusi, J. Cebrecos, A. Jacobs, A. Morte, E. Ortiz, M. Sust, A. Vaqué, I. Gottlieb, S. Videla, N. Gascón, and C. Plata‐Salamán verified the underlying data.

## CONFLICT OF INTEREST

This study was supported by ESTEVE Pharmaceuticals S.A. E.R. Viscusi reports consulting fees for ESTEVE Pharmaceuticals, Fresenius, Heron Therapeutics, Innocoll Pharmaceuticals, Merck, and Salix Pharmaceuticals. O. de Leon‐Casasola reports personal fees for advisory board membership for ESTEVE Pharmaceuticals during the conduct of the study, and for ESTEVE Pharmaceuticals, Stimgenix, and Medtronic outside the submitted work. J. Cebrecos, A. Morte, E. Ortiz, M. Sust, A. Vaqué, and N. Gascón are employees of ESTEVE Pharmaceuticals. A. Jacobs is an employee of Premier Research (Premier Research was paid commercial fees by ESTEVE Pharmaceuticals for work on the study). I. Gottlieb reports grants for ESTEVE Pharmaceuticals for participation as a principal investigator in the current study and personal fees for ESTEVE Pharmaceuticals for consulting projects. S. Daniels is an employee of Optimal Research (Optimal Research was paid commercial fees by ESTEVE Pharmaceuticals for work on the study). M.E. Kuss was an employee of Premier Research during the conduct of the study. S. Videla was an employee of ESTEVE Pharmaceuticals during the conduct of the study. C. Plata‐Salamán was an employee of ESTEVE Pharmaceuticals and has pending or issued patents relevant to CTC. The remaining authors have no conflicts of interest to declare.

## Supporting information


Appendix S1

## Data Availability

ESTEVE Pharmaceuticals S.A. will consider requests for de‐identified patient‐level data and supporting study documents from qualified external researchers. Approval of requests will be at the discretion of ESTEVE Pharmaceuticals S.A. and will depend on the scientific merit of the proposed research and intended use of the data. If approval is granted, a Data Sharing Agreement must be signed and access to data will be provided only if ESTEVE Pharmaceuticals S.A. has legal authority to provide the data and there are no contradictory requirements relating to regulatory filings or reviews. Proposals should be sent to esteve@esteve.com.
