# Supplementary material for: Celecoxib‐tramadol co‐crystal in patients with moderate‐to‐severe pain following bunionectomy with osteotomy: A phase 3, randomized, double‐blind, factorial, active‐ and placebo‐controlled trial
Source: Pain Pract. 2022 Jul 8;23(1):8–22. doi: 10.1111/papr.13136 (PMC10084286; doi:10.1111/papr.13136)

Supplemental digital content

Celecoxib-tramadol co-crystal in patients with moderate-to-severe pain following bunionectomy with osteotomy: a phase 3, randomized, double-blind, factorial, active- and placebo-controlled trial

Eugene R. Viscusi, MD, Oscar de Leon-Casasola, MD, Jesús Cebrecos, MD, Adam Jacobs, PhD, Adelaida Morte, MD, Esther Ortiz, RN, Mariano Sust, MD, Anna Vaqué, MD, Ira Gottlieb, DPM, Stephen Daniels, DO, Joseph S. Gimbel, MD, Derek Muse, MD, Peter Winkle, MD, Michael E. Kuss, BS MT(ASCP), Sebastián Videla, MD, Neus Gascón, MD, Carlos Plata-Salamán, MD

Supplementary methods

Eligibility criteria

Inclusion criteria

Signed consent before study entry; ≥18 years old; scheduled to undergo primary unilateral first metatarsal osteotomy with internal fixation with no additional collateral procedure; male and female individuals were eligible—female patients of child-bearing age were required to be non-lactating and non-pregnant (negative serum pregnancy test at screening and negative urine test on the day before surgery) and to practice effective methods of birth control (hormonal methods, total abstinence from sexual intercourse, intrauterine device, or double-barrier method) from screening until 4 weeks after the last administration of study medication; body weight ≥45 kg and body mass index (BMI) ≤40 mg/m^2; qualifying pain score of ≥5 and ≤9 on the 0 to 10 numerical pain rating scale at rest following cessation of popliteal sciatic block for bunionectomy; good physical health (investigator's judgment); sufficiently alert to communicate with the study observer.

Exclusion criteria

Received any analgesic medication other than short-acting preoperative or intraoperative anesthetic agents before the end of bunionectomy surgical procedure (patients who received any analgesic medication immediately after the bunionectomy surgical procedure was completed and before study medication was administered were also excluded, except for intravenous (IV) ketorolac 30 mg as supplemental analgesia during the continuous infusion period and up until 1:00 AM); history of seizures or alcohol abuse within the past 5 years; history of drug abuse within the past 6 months; history of positive test results for human immunodeficiency virus, hepatitis B, or hepatitis C; active malignancy or diagnosis of cancer within 5 years of screening; currently receiving anticoagulants or antiplatelets (except aspirin ≤325 mg/day); received a course of systemic or intra-articular corticosteroids within 3 months of screening; any ongoing condition capable of confounding assessments of postoperative pain (e.g., severe osteoarthritis of the target joint, fibromyalgia, rheumatoid arthritis, diabetic foot pain or neuropathy, moderate-to-severe headache); receiving or had received chronic (defined as daily use for >2 weeks) opioid therapy (oral codeine, dextromoramide, dihydrocodeine, oxycodone, or morphine-like antitussive) defined as >15 morphine milligram equivalents per day for >3 of 7 days/week over a 1-month period within 12 months of surgery, or had been treated chronically with opioid analgesic (buprenorphine, nalbuphine, or pentazocine) or non-steroidal anti-inflammatory drug (NSAID) within 30 days before screening; received a long-acting NSAID within 4 days before initiation of study medication (except aspirin ≤325 mg/day), or a short-acting NSAID within 1 day, except for IV ketorolac 30 mg as supplemental analgesia during the continuous infusion period; under long-term treatment with opioid agonist/antagonists; use of drugs with enzyme-inducing properties within 3 weeks of surgery (e.g., rifampicin, St. John's Wort, inhibitors/inducers of CYP3A4, CYP2C9, or CYP2D6); pregnant or lactating; any complication during the primary bunionectomy surgery; received monoamine oxidase inhibitors, tricyclic antidepressants, neuroleptics, or other drugs that reduce the seizure threshold, within 4 weeks of study entry; history or evidence of a clinically significant gastrointestinal (GI) event within 6 months before screening, or any history of peptic or gastric ulcers or GI bleeding; clinically significant

renal or hepatic disease, as indicated by clinical laboratory assessment (results ≥3× the upper limit of normal [ULN] for any liver function test, including aspartate aminotransferase, alanine aminotransferase, bilirubin, and lactate dehydrogenase, or creatinine ≥1.5× ULN); clinically significant laboratory or 12-lead electrocardiogram finding at screening that contraindicated study participation (e.g., QTc >450 ms [male] or >470 ms [female]); known history of allergic reaction or clinically significant intolerance to acetaminophen, aspirin, opioids, or any NSAIDs; history of NSAID-induced bronchospasm (patients with the triad of asthma, nasal polyps, and chronic rhinitis are at a greater risk for bronchospasm and were to be considered carefully) or to the ingredients of the study medication, or any other drugs used in the study, including anesthetics and antibiotics that may be required on the day of surgery; received antidepressant medication with serotonin–norepinephrine reuptake inhibitors (milnacipran, duloxetine, venlafaxine), diet pills (including fenfluramine and phentermine), or methylphenidate, or other similar medications for attention-deficit/hyperactivity disorder, within 4 weeks of study entry. Individuals receiving selective serotonin reuptake inhibitors were included provided they had been on a stable dose for 60 days before study participation and planned to remain on that dose throughout the study; any risk in terms of the precautions, warnings, and contraindications in the package insert for tramadol hydrochloride or celecoxib; known coagulation disorder; history of or current medical, surgical, postsurgical, or psychiatric condition that would confound interpretation of safety, tolerability, or efficacy; received an experimental drug or used an experimental medical device within 30 days before screening; undergoing concomitant surgical procedure(s).

Table S1

Sensitivity analyses for the primary efficacy endpoint, $SPID_{0–48}$ (full analysis set).

	LS mean $SPID_{0-48}$	Comparison	LS mean difference	*p*-value[a]
Sensitivity analysis 1: WOCF for dropouts due to adverse events or lack of efficacy				
CTC (*n* = 184)	−148.3	–	–	–
Tramadol (*n* = 183)	−120.1	CTC versus tramadol	−28.2	<0.01
Celecoxib (*n* = 181)	−108.3	CTC versus celecoxib	−40.0	<0.001
Placebo (*n* = 89)	−81.6	CTC versus placebo	−66.7	<0.001
Sensitivity analysis 2: WOCF for all missing data				
CTC (*n* = 184)	−137.2	–	–	–
Tramadol (*n* = 183)	−105.6	CTC versus tramadol	−31.6	<0.001
Celecoxib (*n* = 181)	−98.1	CTC versus celecoxib	−39.2	<0.001
Placebo (*n* = 89)	−67.7	CTC versus placebo	−69.6	<0.001
Sensitivity analysis 3: No imputation for rescue medication use				
CTC (*n* = 184)	−157.9	–	–	–
Tramadol (*n* = 183)	−135.1	CTC versus tramadol	−22.7	<0.01
Celecoxib (*n* = 181)	−127.9	CTC versus celecoxib	−30.0	<0.001
Placebo (*n* = 89)	−106.6	CTC versus placebo	−51.2	<0.001
Sensitivity analysis 4: Adjusted analysis for rescue medication use				
CTC (*n* = 184)	−144.6	–	–	–
Tramadol (*n* = 183)	−133.4	CTC versus tramadol	−11.2	0.15
Celecoxib (*n* = 181)	−134.7	CTC versus celecoxib	−9.9	0.21
Placebo (*n* = 89)	−120.4	CTC versus placebo	−24.1	0.01
Sensitivity analysis 5: Multiple imputation				
CTC (*n* = 183)	−147.3	–	–	–
Tramadol (*n* = 183)	−122.2	CTC versus tramadol	−25.0	<0.01
Celecoxib (*n* = 182)	−113.1	CTC versus celecoxib	−34.2	<0.001
Placebo (*n* = 89)	−86.5	CTC versus placebo	−60.8	<0.001

Abbreviations: CTC, celecoxib-tramadol co-crystal; LS, least-squares; $SPID_{0-48}$, sum of pain intensity difference to 48 h; WOCF, worst observation carried forward.

[a] *p*-values derived from analysis of covariance, adjusting for center and baseline pain intensity.

Table S2.

Responder analyses (full analysis set).

	**Treatment group, n (%)**				**Odds ratio (95% CI)**		
	CTC (n = 184)	Tramadol (n = 83)	Celecoxib (n = 81)	Placebo (n = 89)	CTC versus tramadol	CTC versus celecoxib	CTC versus placebo
Responders 50%[a,b]	115 (62.5)	107 (58.5)	107 (59.1)	49 (55.1)	1.204 (0.777, 1.864)	1.167 (0.752, 1.811)	1.377 (0.806, 2.353)
Responders 30%[a,c]	128 (69.6)	118 (64.5)	128 (70.7)	58 (62.5)	1.303 (0.820, 2.070)	0.937 (0.582, 1.507)	1.230 (0.696, 2.174)
Responders NRS <4*[,d]	117 (63.6)	108 (59.0)	109 (60.2)	49 (55.1)	1.245 (0.800, 1.939)	1.160 (0.743, 1.811)	1.440 (0.838, 2.473)
Responders 50% and NRS <4[a]	113 (61.4)	105 (57.4)	105 (58.0)	46 (51.7)	1.202 (0.778, 1.855)	1.164 (0.752, 1.800)	1.509 (0.887, 2.569)
Responders 30% and NRS <4[a]	117 (63.6)	108 (59.0)	109 (60.2)	49 (55.1)	1.245 (0.800, 1.939)	1.160 (0.743, 1.811)	1.440 (0.838, 2.473)

Abbreviations: CI, confidence interval; CTC, celecoxib-tramadol co-crystal; NRS, numerical rating scale.

[a] Logistic regression adjusted for center and baseline pain.

[b] A 50% reduction in pain intensity from baseline sustained until the end of the 48-h observation period.

[c] A 30% reduction in pain intensity from baseline sustained until the end of the 48-h observation period.

[d] A pain intensity below 4 on the NRS sustained until the end of the 48- h observation period.

Figure S1. Placebo-adjusted analysis of the primary efficacy endpoint, $SPID_{0-48}$ (post hoc analysis). Analyzed using analysis of covariance, adjusting for center and baseline pain intensity. CI, confidence interval; CTC, celecoxib-tramadol co-crystal; SPID, sum of pain intensity differences

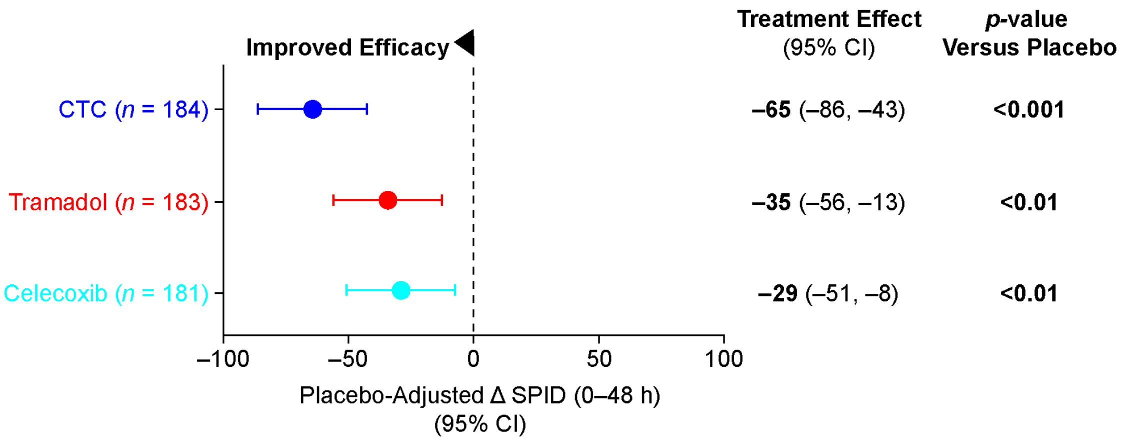

Figure S2. Subgroup analyses of the primary efficacy endpoint, SPID$_{0\text{-}48}$. BMI, body mass index; CI, confidence interval; CTC, celecoxib-tramadol co-crystal; SPID, sum of pain intensity differences

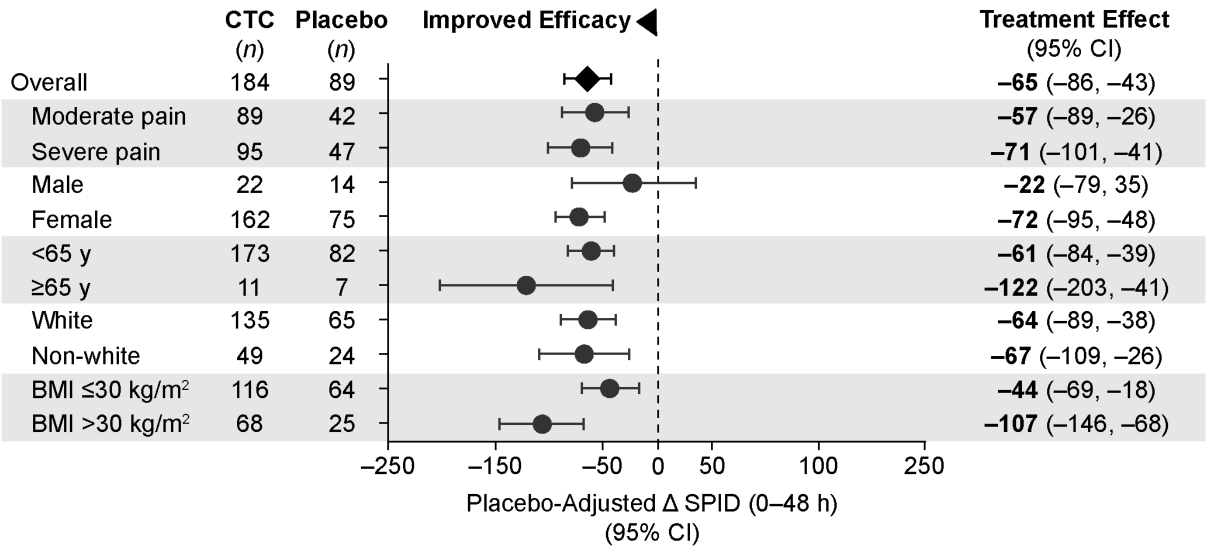

Supplement: Supplementary file 1 — Appendix S1 [file PAPR-23-8-s001.pdf]
